# Dietary Selenium Intake and Subclinical Hypothyroidism: A Cross-Sectional Analysis of the ELSA-Brasil Study

**DOI:** 10.3390/nu10060693

**Published:** 2018-05-30

**Authors:** Gustavo R. G. Andrade, Bartira Gorgulho, Paulo A. Lotufo, Isabela M. Bensenor, Dirce M. Marchioni

**Affiliations:** 1 Department of Nutrition, School of Public Health, University of São Paulo, São Paulo CEP 03178-200, Brazil; gustavoandrade@usp.br; 2 Department of Food and Nutrition, School of Nutrition, Federal University of Mato Grosso, Cuiabá CEP 78060-900, Brazil; bartira.gorgulho@gmail.com; 3 Clinical and Epidemiological Research Center, University Hospital, University of São Paulo, São Paulo CEP 05508-000, Brazil; palotufo@usp.br (P.A.L.); isabensenor@gmail.com (I.M.B.)

**Keywords:** selenium, diet, subclinical hypothyroidism, adults, thyroid

## Abstract

Selenium (Se) participates in several enzymatic reactions necessary for regulating the homeostasis of thyroid hormones. We aimed to analyze the association between dietary Se intake and subclinical hypothyroidism. Baseline data from the Longitudinal Study of Adult Health (Estudo Longitudinal de Saúde do Adulto—ELSA-Brasil) in Brazil were analyzed, with a final sample size of 14,283 employees of both sexes aged 35–74 years. Dietary data was collected using a previously validated food frequency questionnaire. Subclinical hypothyroidism was categorized as thyroid-stimulating hormone levels of >4.0 IU/mL and free prohormone thyroxine levels within normal limits, without administering drugs for thyroid disease. A multiple logistic regression model was used to assess the relationship between the presence of subclinical hypothyroidism and tertiles of Se consumption. The prevalence of subclinical hypothyroidism in the study sample was 5.4% (95% confidence interval [CI], 3.8–7.0%). Compared with the first tertile of Se intake, the second (odds ratio [OR], 0.79; 95% CI, 0.65–0.96%) and third (OR, 0.72; 95% CI, 0.58–0.90%) tertiles were inversely associated with subclinical hypothyroidism, however further research is needed to confirm the involvement of Se in subclinical hypothyroidism using more accurate methodologies of dietary assessment and nutritional status to evaluate this relationship.

## 1. Introduction

One of the diseases that affect the thyroid gland is subclinical hypothyroidism, which is characterized by elevated serum levels of thyroid-stimulating hormone (TSH) at a concentration recommended for prohormone thyroxine (T4) and active hormone triiodothyronine (T3). The decompensated levels of thyroid hormones may contribute to atherosclerotic events [1] and an increase in cardiovascular-related mortality [2]. Also, observational longitudinal studies have shown an inverse association between selenium exposure and risk of some cancer types but still to be confirmed [3]. It is estimated that subclinical hypothyroidism affects 3–8% of the general population and is more common in women than in men [4]. In Brazil, an epidemiological study in elderly reported that prevalence of subclinical hypothyroidism was 6.5% [5]. Olmos et al. [6], in the Brazilian Longitudinal Study of Adult Health (ELSA-Brasil), reported that subclinical hypothyroidism prevalence was 5.4% overall.

Hypothyroidism is sometimes difficult to diagnosis, since most of the symptoms, such as fatigue, lack of concentration, dry skin, are nonspecific and frequently attributed to other causes or to the aging process itself [2]. Studies conducted in Brazil demonstrated the influence of race on the prevalence of hypothyroidism, which was lower in black and brown people [6,7]. Also, gender, race and socioeconomic status were reported to influence the diagnosis and treatment of hypothyroidism, with men, browns, blacks and subjects with low socioeconomic status having lower frequencies of treatment for hypothyroidism [6]. 

The thyroid gland contains high levels of selenium (Se) [8] and expresses a variety of selenoproteins that are involved in protection of oxidative stress and metabolism of thyroid hormones (TH) [9,10,11]. Se deficiency impairs regular synthesis of selenoproteins and adequate TH metabolism. However, on selenium deficient diets, endocrine organs and the brain are preferentially supplied [12], especially the thyroid gland, that retains the trace element very efficiently [11,13]. On the other hand, Parshukova et al. [14], studying the interrelationships between seasonal selenium levels and levels of thyroid gland hormones over a year, verified that low levels of plasma selenium affected thyroid hormone levels in humans living in North European Russia. Wu et al. [15] reported in a study on China that higher serum selenium was associated with lower chance to present subclinical hypothyroidism. 

Se nutritional status varies worldwide because the Se content in food is related to the amount in the soil [16]. Thus, the plasma Se concentrations are variable in different populations around the world. For instance, plasma Se is higher in the USA compared to the South Islands of New Zealand [17]. A study in São Paulo, Brazil, using biomarkers of Se status, reported that plasma Se concentrations were very low compared with those observed in other healthy populations, such as the USA, New Zealand and UK [18]. They hypothesized that, as Se intake can be predicted by plasma Se concentrations, this lower concentration could be a consequence of low Se intake and the low Se content in foods in this southern region of Brazil. According to a study conducted by Favaro et al. [19], the food intake of selenium in Brazil can vary from 20 to 114 μg/day, that is, from low to adequate, depending on the region that was studied and the socioeconomic level of the population. Usually the main sources of Se are cereals, meats and fish [20]. Ferreira et al. [21], evaluated the selenium content in foods consumed in different states of Brazil and the ingredients that are considered staple food, such as beans, wheat flour, rice, cassava flour and maize, were poor sources of selenium, while animal sources, more expensive, were better sources. 

Despite the expected relationship between Se and thyroid function, only one [22] of several studies [22,23,24,25,26], which evaluated thyroid metabolism in different populations, found a positive effect of Se supplementation on thyroid hormone levels.

The objective of this study was to analyze the association between the dietary intake of Se and subclinical hypothyroidism based on baseline data from the Longitudinal Study of Adult Health (Estudo Longitudinal de Saúde do Adulto—ELSA-Brasil). 

## 2. Materials and Methods

This cross-sectional study analyzed baseline data from the ELSA study in Brazil, a multicenter cohort study focused on chronic diseases, particularly cardiovascular diseases and comprised 15,105 employees from six Brazilian institutions of higher education and research aged 35–74 years.

Baseline data were collected from 2008 to 2010 by conducting interviews to identify sociodemographic, lifestyle, anthropometric, dietary and clinical characteristics. Details of the study design were reported by Aquino et al. [27] and Bensenor et al. [28].

**Figure 1:**
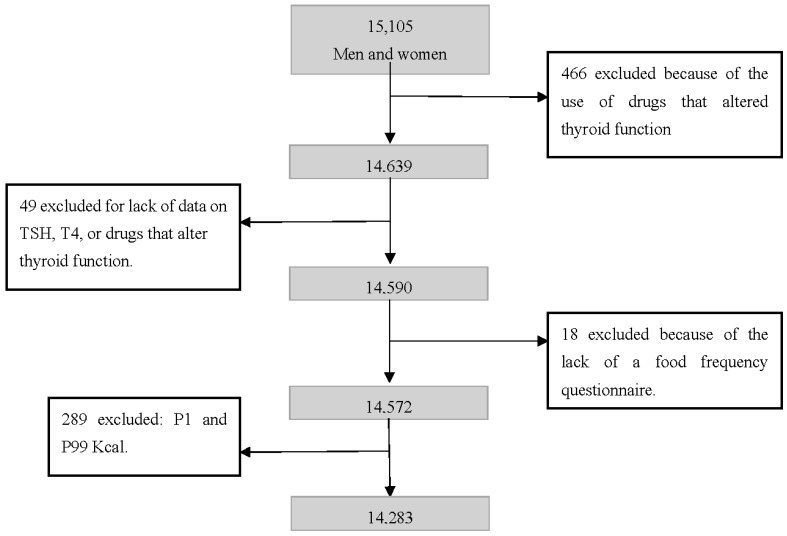
Exclusion criteria and final sample. ELSA-Brasil, Brazil 2017.

The study sample consisted of 14,283 participants. The exclusion criteria were the use of drugs that modified thyroid function, lack of information on TSH and T4, absence of a food frequency questionnaire (FFQ) and energy intake lower than the first percentile or higher than the 99th percentile of the distribution (Figure 1).

### 2.1. Ethical Aspects

The ELSA-Brasil protocol was approved at all 6 centers: Oswaldo Cruz Foundation (Fiocruz), Federal University of Bahia (UFBA), Federal University of Espírito Santo (UFES), Federal University of Minas Gerais (UFMG), Federal University of Rio Grande do Sul (UFRGS) and University of São Paulo (USP), by the institutional review boards addressing research in human participants. All participants signed a written informed consent form.

### 2.2. Diet

Food intake was obtained using a validated FFQ with 114 food items to evaluate diet in the past 12 months [29], covering three sections: food products/food preparations, measures of consumed products and consumption frequencies with eight response options: “more than 3 times a day”, “2 to 3 times a day”, “once a day”, “5 to 6 times a week”, “2 to 4 times a week”, “once a week”, “1 to 3 times a month” and “never/rarely”. The measures of consumed foods were determined using a toolkit [30].

### 2.3. Subclinical Hypothyroidism

Venous blood was withdrawn from the ELSA participants after a 12-h fast and dosing of TSH. The levels of free T4 were analyzed in participants with low TSH (<0.4 IU/mL) or high TSH (>4.0 IU/mL). TSH and FT4 were measured using a third-generation immunoenzymatic assay (Siemens, Deerfield, IL, USA) in serum obtained from centrifuged venous blood samples after overnight fasting [31]. FT4 levels were measured in participants exhibiting altered TSH levels. In this study, reference range levels were 0.4–4.0 mIU/L for TSH and 10.3–24.45 pmol/L for FT4. We excluded participants using drugs that could interfere with thyroid function: amiodarone, carbamazepine, carbidopa, phenytoin, furosemide, haloperidol, heparin, interferon, levodopa, lithium, metoclopramide, propranolol, primidone, rifampicin and valproic acid. 

ELSA-Brasil study participants were classified into five categories of thyroid function, according to TSH and FT4 levels and information related to the use of medication to treat thyroid disorders: clinical hyperthyroidism (low serum TSH and high FT4 levels or use of medication to treat hyperthyroidism), subclinical hyperthyroidism (low serum TSH, normal FT4 levels and no use of drugs to treat thyroid diseases), euthyroidism (normal TSH and no use of thyroid drugs), subclinical hypothyroidism (high TSH levels, normal FT4 levels and no use of drugs to treat thyroid diseases) and clinical hypothyroidism (high TSH and low FT4 levels, or use of levothyroxine to treat hypothyroidism). For the descriptive analysis all types were included, but, only participants with subclinical hypothyroidism or euthyroidism were included on the regression models.

The cutoff points used to determine subclinical hypothyroidism were TSH levels of >4.0 IU/mL with free T4 within the recommended doses, without the use of drugs that alter thyroid function.

### 2.4. Statistical Analysis

Multiple logistic regression models with nutrients adjusted for total energy, using the residuals method [32], were conducted in the sample that included only participants with subclinical hypothyroidism or euthyroidism. The models were adjusted for age (35–59 years, ≥60 years), sex (male and female), nutritional status (body mass index) in kg/m² (low weight, eutrophic, overweight and obese according to the cut-off points recommended by the World Health Organization) [33], smoking (no for ex-smokers and non-smokers and yes for smokers), hypertension (yes or no; obtained from systolic blood pressure ≥140 mmHg and/or diastolic blood pressure ≥90 mmHg, or use of drugs for treating hypertension), diabetes (yes or no, obtained from data on post-prandial glycaemia, glycated hemoglobin, use of medications for treating diabetes and previous diagnosis of diabetes), dyslipidemia (yes or no, obtained from previous diagnosis of the disease and use of medicines), per capita income (obtained from data on the net family income of the past month, by the average of extreme values of each category and number of family members who depended on this income to live), current alcohol use (yes or no), level of physical activity during leisure (low, moderate, or high) according to the International Physical Activity Questionnaire (IPAQ), change in diet (yes or no) and use of dietary supplements (regularly or not). 

Micronutrients that correlated with the outcome of interest and thyroid function, including zinc, vitamin A, iodine and sodium, were also used as adjustment variables [34]. Urinary sodium (g/day) was used as a proxy for iodine consumption [35].

All analyses were performed using Stata Statistical Software (release 14, 2015, StataCorp LP, College Station, TX, USA) and the level of significance was set at 5%. 

## 3. Results

The total sample had a higher proportion of participants who were Caucasian, female, aged 35–59 years, with per capita income in the first tertile, non-smokers, alcohol users, with low physical activity level during leisure, without significant changes in diet, overweight, non-hypertensive, non-diabetic, dyslipidemic and euthyroid (Table 1).

The major food sources of dietary selenium verified in this study were: rice (23%), meat (13%), bread (12%), beans (10%), milk (10%), fish (8%), pasta (5%) and nuts (4%).

The lower tertile of Se consumption had a higher proportion of participants who were males, of Black and mixed race, aged 35–59 years, in the lowest tertile of per capita income, non-smokers, alcohol consumers, with a low level of physical activity during leisure, without significant changes in diet, not using dietary supplements, overweight, non-hypertensive, non-diabetic, dyslipidemic and euthyroid. The highest tertile of Se intake had a predominance of participants who were Caucasian, female, aged 39–59 years, in the highest tertile of per capita income, non-smokers, alcohol users, with a low level of physical activity during leisure, without significant changes in the diet, not using food supplements, non-hypertensive, non-diabetic, dyslipidemic and euthyroid (Table 1).

The analysis of the other nutrients showed a correlation with thyroid function with respect to the consumption tertiles relative to Se intake tertiles (Table 2). All analyzed micronutrients were positively correlated with Se intake, particularly total fats, which presented a higher correlation coefficient (r = 0.33) (Table 2).

Table 3 shows the inverse association between Se intake and subclinical hypothyroidism based on logistic regression models adjusted for gender, self-reported race, age, per capita income, current smoking, current alcohol use, physical activity, use of supplements, dietary change in the past 6 months, total energy intake, total and saturated fat consumption, zinc and vitamin A consumption, urinary sodium, nutritional status, diabetes, hypertension and dyslipidemia.

## 4. Discussion

Selenium intake showed an inverse association with subclinical hypothyroidism, independent of the intake of energy and other nutrients that were previously shown to be correlated with thyroid function.

Most studies that analyzed the effects of Se on thyroid function and hypothyroidism are experimental and three studies [36,37,38] assessed the effect of Se supplementation on the enzymatic and hormonal functions essential for thyroid maintenance in Se-deficient rats. These studies found a positive correlation between Se and the analyzed parameters. Understanding the health implications of Se in humans has been far more difficult, as Se intakes nor tissue levels of free-living people can seldom be ascertained with the levels of confidence typical of controlled animal experiments [17].

It is well known that the thyroid gland retains selenium and selenoprotein activity even under conditions of severe deficiency [39]. However, it remains unknown whether selenium modulates peripheral thyroid hormone action via less prioritized mechanisms [13]. A cross-sectional study [15] involving 6152 participants from two municipalities in China determined the prevalence of thyroid diseases in two similar areas, except in participants with extreme Se levels and showed that low selenium status was associated with increased risk of thyroid disease and concluded that increased selenium intake may reduce the risk in areas of low selenium intake. However, Thomson et al. [40] analyzed data from two cross-sectional and three interventional studies conducted in New Zealand on the effects of Se on thyroid metabolism and found no significant correlations, even after Se supplementation. 

Despite data on large controlled trials, that would provide more reliable evidence, are scarce, randomized controlled trials, conducted in healthy and diseased participants and used different doses of supplemental Se, found no significant effects of this micronutrient on the outcome of interest [41,42,43,44]. Studies that reported detrimental [45,46] or worsening results also were found in literature [47]. Despite that, in general, an improvement on levels of Se were observed [45,48,49]. In a prospective randomized controlled study by Pirola et al. [50] involving 192 patients supplemented with Se for 4 months, euthyroidism was restored in 1/3 of subclinical hypothyroidism patients with autoimmune thyroiditis.

Despite the lack of studies and inconclusive evidence, Negro et al. [51] (2016) evaluated a sample of 778 Italian endocrinologists and observed that more than two-thirds of the study population used Se supplementation as a therapy for subclinical hypothyroidism and 60% of this sample suggested daily doses of 100–200 μg, 20–30% recommended doses of <100 μg and 10–20% suggested doses of >200 μg. These recommendations are higher than those proposed by the Dietary Reference Intakes [52].

The present study has limitations. Our estimates of selenium intake were obtained from a FFQ, a method that is widely used in large epidemiological studies to determine the frequency of consumption of specific food products in 1 year [29]. This method is appropriated to rank individual according to levels of intake, however, it is not considered the most appropriate for the quantitative analysis of micronutrients because it tends to overestimate dietary intake and. Moreover, as it lacks accuracy, does not allow the evaluation of the adequacy of the ingested micronutrients within this period, therefore only allowing a comparison between major and minor consumers. Nutrient intake can be better estimated using repeated 24-h recalls and dietary records because the mean values obtained from several days of dietary intake yield safer and more reliable results [53]. However, as these methods are based on the individual report, they still are susceptible to bias. For greater accuracy of the selenium levels in the participants, it would be necessary to use biomarkers capable of indicating more precisely the condition of these individuals [3,12,20]. Especially taking into account that there is enormous variability of Se levels in foods, as it is dependent exclusively on the soil properties from which they were harvested. So, the demonstrated selenium consumption tertiles may not correspond to the participants’ exact selenium levels. However, we used the obtained Se estimates to rank individual according to their intake and we used the distribution of this intake in our population to define cutoffs. We used the same composition table that was used in the last National Dietary Survey 2009–2009 in Brazil, carefully checked for the completeness of Se information. In this case, we expect a systematic bias and a hypothesized relationship is possible to identify, that need to be confirmed. Another important limitation lies in the design of the study, since it is a cross-sectional observational study and suffer from limitations inherent to the observational design, including exposure misclassification and unmeasured confounding. Although it has been adjusted by several dietary and non-dietary factors, these are data from ELSA-Brasil baseline and we did not longitudinally evaluate food consumption in this population, which would be more appropriate. Therefore, because it is an observational study, the results found on the relationship between selenium and the outcome cannot be interpreted as a causal relation, requiring that they would be confirmed in further studies. 

## 5. Conclusions

The results revealed an inverse correlation between Se intake and subclinical hypothyroidism. However, further research is needed to confirm the involvement of Se in subclinical hypothyroidism using more accurate methodologies of dietary assessment and nutritional status to evaluate this relationship.

## Figures and Tables

**Table 1:** Description of the total population and selenium consumption per tertile in the ELSA-Brasil study, 2017.

	Total		Selenium Intake *						
			First Tertile (0–187 mg)		Second Tertile (188–232 mg)		Third Tertile (233–1087 mg)		*p* Value **
	N	%	N	%	N	%	N	%	
Sex									<0.001
Male	6518	45.6	2519	53.0	1979	41.6	2020	42.4	
Female	7765	54.4	2242	47.0	2782	58.4	2741	57.6	
Self-declared race									<0.001
Caucasian	7418	52.5	2045	43.4	2540	53.9	2833	60.3	
Black and Mixed	6204	43.9	203	53.1	190	42.3	111	36.4	
Others	497	3.6	161	3.5	179	3.8	157	3.3	
Age									<0.001
35–59 years	11,271	78.9	3892	80.5	3831	80.5	3548	74.5	
≥60 years	3012	21.1	869	18.6	930	19.5	1213	25.5	
Per capita income									<0.001
First tertile (USD 14.85–520.51)	5175	36.1	2498	52.7	1665	35.1	1012	21.3	
Second tertile (USD 529.87–1059.74)	4992	34.9	1476	31.1	1739	36.6	1707	36.0	
Third tertile (USD 1115.32–4238.97)	4135	29.0	768	16.2	1342	28.3	2025	42.7	
Current smoking									<0.001
No	12,446	87.1	3979	83.6	4182	87.9	4285	90.0	
Yes	1836	12.9	782	16.4	578	12.1	476	10.0	
Current alcohol use									<0.001
No	4302	30.1	1675	35.2	1434	30.1	1193	25.1	
Yes	9978	69.9	3085	64.8	3325	69.9	3568	74.9	
Physical activity during leisure									<0.001
Low	10,796	76.7	3865	82.6	3621	77.3	3310	70.3	
Moderate	1986	14.1	499	10.7	683	14.6	804	17.2	
Vigorous	1287	9.2	315	6.7	379	8.1	593	12.5	
Change in diet									<0.001
No	9903	69.4	3517	73.9	3247	68.3	3139	66.0	
Yes	4366	30.6	1243	26.1	1507	31.7	1616	34.0	
Use of dietary supplements									<0.001
No	10,887	77.3	3940	84.2	3639	77.5	3308	70.5	
Regularly	1823	12.9	391	8.3	579	12.3	853	18.0	
Not regularly	1381	9.8	353	7.5	480	10.2	548	11.5	
Nutritional status									<0.001
Low weight	129	0.9	57	1.2	34	0.7	38	0.8	
Eutrophic	5175	36.2	1672	35.1	1662	34.9	1841	38.7	
Overweight	5740	40.2	1898	39.9	1940	40.8	1902	40.0	
Obese	3234	22.7	1,32	23.8	1124	23.6	978	20.5	
Hypertension									<0.001
No	9930	69.5	3047	64.0	3120	65.5	3163	66.4	
Yes	4951	34.7	1714	36.0	1640	34.5	1597	33.6	
Diabetes									<0.001
No	11,558	80.9	3797	79.8	3886	81.6	3875	81.4	
Yes	2724	19.1	963	20.2	875	18.4	886	18.6	
Dyslipidemia									<0.001
No	6007	42.4	2225	47.0	1926	40.8	1856	39.4	
Yes	8169	57.6	2510	53.0	2799	59.2	2860	60.6	
Thyroid function									<0.001
Subclinical hypothyroidism	770	5.4	276	5.8	252	5.3	242	5.1	
Clinical hypothyroidism	1061	7.4	256	5.4	383	8.0	422	8.9	
Euthyroid	12,171	85.3	4146	87.1	4022	84.5	4003	84.1	
Subclinical hyperthyroidism	186	1.3	57	1.2	70	1.5	59	1.2	
Clinical hyperthyroidism	95	0.6	26	0.5	34	0.7	35	0.7	

* Energy-adjusted nutrient; ** *p* values of the chi-square test.

**Table 2:** Intake of energy and micronutrients and urinary sodium per selenium intake tertile in the ELSA-Brasil study, 2017.

	Total	Selenium Intake *							R ***
		First Tertile (0–187 mg)		Second Tertile (188–232 mg)		Third Tertile (233–1087 mg)		*p* Value **	
	N	N	%	N	%	N	%		
Energy (average)								<0.001	_
First tertile (1900 kcal)	4761	1049	22.0	2202	46.2	1510	31.7		
Second tertile (2735 kcal)	4761	1599	33.6	1529	32.1	1633	34.3		
Third tertile (4166 kcal)	4761	2113	44.4	1030	21.6	1618	34.0		
Zinc * (average)								<0.001	0.16
First tertile (13 mg)	4761	2562	53.8	1226	25.7	973	20.4		
Second tertile (16 mg)	4761	1278	26.8	1833	38.5	1650	34.7		
Third tertile (21 mg)	4761	921	19.3	1702	35.8	2138	44.9		
Vitamin A * (average)								<0.001	0.11
First tertile (71 mg)	4761	1887	39.6	1614	33.9	1260	26.5		
Second tertile (125 mg)	4761	1443	30.3	1714	36.0	1604	33.7		
Third tertile (220 mg)	4761	1431	30.1	1433	30.7	1897	40.6		
Total fat * (average)								<0.001	0.33
First tertile (74 g)	4761	2335	49.0	1418	29.8	1008	21.2		
Second tertile (93 g)	4761	1399	29.4	1815	38.1	1547	31.6		
Third tertile (112 g)	4761	1027	21.6	1528	32.1	2206	47.2		
Saturated fat * (average)								<0.001	0.13
First tertile (22 g)	4761	2107	44.3	1354	28.4	1300	27.8		
Second tertile (30 g)	4761	1315	28.1	1825	39.1	1621	34.7		
Third tertile (41 g)	4761	1339	28.7	1582	33.9	1840	39.0		
Urinary sodium (average)								<0.001	−0.08
First tertile (6 g/day)	4665	1345	28.8	1537	33.0	1773	38.1		
Second tertile (10 g/day)	4667	1528	32.8	1563	33.6	1576	33.9		
Third tertile (19 g/day)	4642	1782	38.4	1557	33.4	1303	28.0		

* Energy-adjusted nutrient; ** *p* value of the chi-square test; *** Pearson correlation coefficient between selenium and other nutrients (*p* < 0.001).

**Table 3:** Logistic regression models between subclinical hypothyroidism (outcome)* and selenium intake adjusted for the consumption of zinc, vitamin A, total and saturated fats and urinary sodium. ELSA-Brasil study, 2017.

	Model 1 ^a^			Model 2 ^b^			Model 3 ^c^			Model 4 ^d^			Model 5 ^e^		
	OR	95% CI		OR	95% CI		OR	95% CI		OR	95% CI		OR	95% CI	
Selenium **															
First tertile (0–187 mg)	1	_	_	1	_	_	1	_	_	1	_	_	1	_	_
Second tertile (188–232 mg)	0.89	0.74	1.08	0.85	0.71	1.03	0.81	0.67	0.98	0.80	0.66	0.97	0.79	0.65	0.96
Third tertile (233–1087 mg)	0.90	0.75	1.08	0.86	0.70	1.05	0.74	0.60	0.92	0.73	0.59	0.91	0.72	0.58	0.90

* For this analysis the sample includes only participants with subclinical hypothyroidism or euthyroidism; ** Energy-adjusted nutrients using the residuals method; ^a^ Model used to assess the correlation between subclinical hypothyroidism and selenium intake tertiles adjusted for energy intake, use of dietary supplements and diet change in the past 6 months; ^b^ Model adjusted by model 1 variables plus, zinc, vitamin A, total and saturated fats and urinary sodium; ^c^ Model adjusted by model 2 variables plus gender, age, per capita income and self-declared race; ^d^ Model adjusted by model 3 variables plus current smoking, current alcohol use and practice of physical activity; ^e^ Model adjusted by model 4 variables plus presence of diabetes, arterial hypertension, dyslipidemia and nutritional status.
